# Diagnostic biopsy does not accurately reflect the PD-L1 expression in triple-negative breast cancer

**DOI:** 10.1007/s10238-023-01190-2

**Published:** 2023-10-07

**Authors:** Marek Zdrenka, Adam Kowalewski, Jędrzej Borowczak, Joanna Łysik-Miśkurka, Hanna Andrusewicz, Tomasz Nowikiewicz, Łukasz Szylberg

**Affiliations:** 1 Department of Tumor Pathology and Pathomorphology, Oncology Center, Prof. Franciszek Łukaszczyk Memorial Hospital, Bydgoszcz ul. Romanowska, 85-796 Bydgoszcz, Poland; 2 Department of Obstetrics, Gynaecology and Oncology, Chair of Pathomorphology, Placentology, and Clinical Hematopathology, Collegium Medicum in Bydgoszcz, Nicolaus Copernicus University in Torun, Torun, Poland; 3 Clinical Department of Breast Cancer and Reconstructive Surgery, Oncology Center, Prof. Franciszek Łukaszczyk Memorial Hospital, Bydgoszcz, Poland; 4 Department of Surgical Oncology, Collegium Medicum in Bydgoszcz, Nicolaus Copernicus University in Torun, Bydgoszcz, Poland

**Keywords:** Cancer, PD-L1, Triple-negative breast cancer, Pathology, Diagnosis

## Abstract

PD-L1 expression is known to predict the benefits of immune checkpoint inhibitor therapy for triple-negative breast cancer (TNBC). We examined whether the PD-L1 expression evaluated in biopsy specimens accurately reflects its expression in the whole tumor. Immunohistochemistry was performed on 81 biopsy and resection specimens from patients with TNBC to determine their PD-L1 status. We found PD-L1-positive tumors in 23 (28%) biopsy specimens and primarily PD-L1-negative tumors in 58 (72%). The PD-L1 status was reevaluated in matching postoperative specimens of primarily PD-L1-negative tumors. Of them, 31% (18/58) were positive, whereas 69% (40/58) were negative. Considering the pre- and postoperative analyses, 41 (51%) patients had PD-L1-positive tumors, while 40 had PD-L1-negative tumors. We found 18 (22%) more PD-L1-positive tumors while examining the resection specimens compared to biopsies, and the difference was statistically significant (*p* = 0.0038). Diagnostic biopsies do not fully reflect the PD-L1 expression in TNBC. Our results suggest that a significant subset of TNBC patients may be misclassified as PD-L1-negative and disqualified from anti-PD-L1 therapy.

## Introduction

Triple-negative breast cancer (TNBC) is a highly aggressive subtype of breast cancer, which accounts for 15–20% of all breast carcinomas. It is associated with a particularly aggressive disease course and a high mortality rate [1, 2]. “Triple negative” refers to the lack of estrogen receptors (ER), progesterone receptors (PgR), and human epidermal growth factor receptor 2 (HER2) on the surface of TNBC cells. Therefore, TNBC patients do not benefit from hormonal and anti-HER2 therapy [3].

Recently, the blockade of the programmed cell death-1/programmed cell death ligand-1 (PD-1/PD-L1) interactions has emerged as a frontline approach to treating TNBC patients. PD-1 (CD279) is a transmembrane glycoprotein present primarily on immune cells, while its ligand, PD-L1, is expressed by tumor and antigen-presenting cells [4]. The binding between PD-1 and PD-L1 initiates apoptotic signaling, leading to lymphocyte exhaustion [4, 5]. Since PD-L1 upregulation facilitates cancer cell immune escape, targeting the PD-L1/PD-1 pathway has become a critical therapy point [6].

The efficacy of anti-PD-1/anti-PD-L1 antibodies depends on the expression of PD-L1 in a tumor. Therefore, in many indications, the tumor PD-L1 status must be checked before the therapy can be started. [7]. Accurate assessment of patients’ PD-L1 status may identify patients who respond to immunotherapy and prevent unnecessary side effects in non-responders [7]. However, available detection methods possess limitations that must be addressed. There are four FDA-approved PD-L1 IHC staining protocols and many IHC tests whose results are not always equivalent [8]. The cutoffs determining whether a tumor is PD-L1-positive or PD-L1-negative differ between cancers and can be determined using various scoring methodologies. High intra-tumor heterogeneity may diminish the viability of PD-L1 assessment, especially when small samples are concerned [9]. To date, the concordance of PD-L1 expression between biopsy and matched postoperative specimens was best investigated in non-small lung carcinoma (NSCLC) and varied among different studies [10, 11]. Those results raise concern for potential patients’ PD-L1 status misclassification, which can impact their chances of receiving adequate therapy.

In this study, we examined whether the PD-L1 expression evaluated by core biopsy accurately reflects its expression in TNBC compared to postresection specimens.

## Material and methods

### Materials

The retrospective study was performed on 98 patients with histologically confirmed TNBC tissue samples who underwent diagnostic trials and surgical treatment. Seventeen patients were excluded due to insufficient material (less than 50 viable tumor cells) or extensive necrotic changes within surgical biopsy material. Eighty-one biopsies and 81 matching surgical specimens were analyzed (Table 1). Assuming that the final PD-L1 status is positive or negative, *p* < 0.05%, and the size of our cohort is 81 samples, the power analysis of the two-group contingency chi-square test reached 98.36%, confirming the validity of the further analysis.

**Table 1:** Patients and TNBC characteristics

No. of patients	81	
Mean age	58 years (32–85 range)	
Tumor size	cT1	21
	cT2	57
	cT3	1
	cT4	3
Nodal status	pN0	56
	pN1	13
	pN2	8
	pN3	4

All biopsies were core needle biopsies. In each case, 2 to 4 core biopsies (average of 3) were performed. The sample was considered positive for PD-L1 expression if at least one biopsy had IC score ≥ 1%. Only biopsies of sufficient size (> 1 cm length) and with preserved histological structure were analyzed to ensure adequate sampling quality.

### PD-L1 immunohistochemistry

All of the collected tissue samples were processed following the standard diagnostic protocol. The specimens were fixed in 10% buffered formalin for 24 h at room temperature. After fixation, the sections were dehydrated in ethyl alcohols (80–99.8%), cleared in xylenes (I–IV), and embedded in paraffin. Then, a preliminary evaluation of tissue samples according to hematoxylin and eosin staining was performed by two independent pathologists. For this study, representative material from tumors (diagnostic biopsy) was selected for routine (ER, PR, HER2) and additional immunohistochemical studies. The immunohistochemical studies of PD-L1 expression were performed using an anti-PD-L1 antibody (clone SP142, Ventana Medical Systems) on material from diagnostic biopsies and matching postoperative specimens. For the immunohistochemical staining, the original protocol provided by Ventana was used. Finally, the brown color due to the histochemical reaction product was considered as observed in the site of the presence of the searched antigen.

### Image analysis

The pathologists evaluating the immunohistochemical expression of the examined antigen worked independently and were blinded to the patient’s data and tissue characteristics. The protein expression was evaluated using a light microscope at 20× original objective magnification. Briefly, the PD-L1 expression in immune cells (IC) score was calculated as the percentage of PD-L1-positive mononuclear tumor-infiltrating immune cells (lymphocytes, macrophages, dendritic cells) at any intensity within the tumor area, including the intratumoral and contiguous peritumoral stroma. Membranous staining of tumor cells was not taken into account. The case was positive if the tissue sample exhibited ≥ 1% of tumor-infiltrating immune cells with PD-L1 expression. All biopsy specimens were initially evaluated and divided into positive and primarily negative based on the expression of PD-L1. Some samples showed a slight PD-L1 staining and, after careful analysis, did not exceed 1% of IC. Those samples were considered negative per assessment criteria and labeled as < 1% IC. Next, the matched surgically resected specimens were used to reevaluate the PD-L1 status in the primarily negative cases. Once again, the patients were categorized as either positive or negative.

### Data analysis

All statistical analyses were performed using Statistica version 13.1 and Microsoft Excel 2019. The *p* value < 0.05 was considered statistically significant. Cohen’s Kappa and percentage agreement rate were used to calculate the agreement rate between biopsy and resection specimen results. For nominal and dichotomous data, the Spearman correlation coefficient was calculated.

## Results

### The analysis of PD-L1 expression in biopsy and resection specimens

**Fig. 1:**
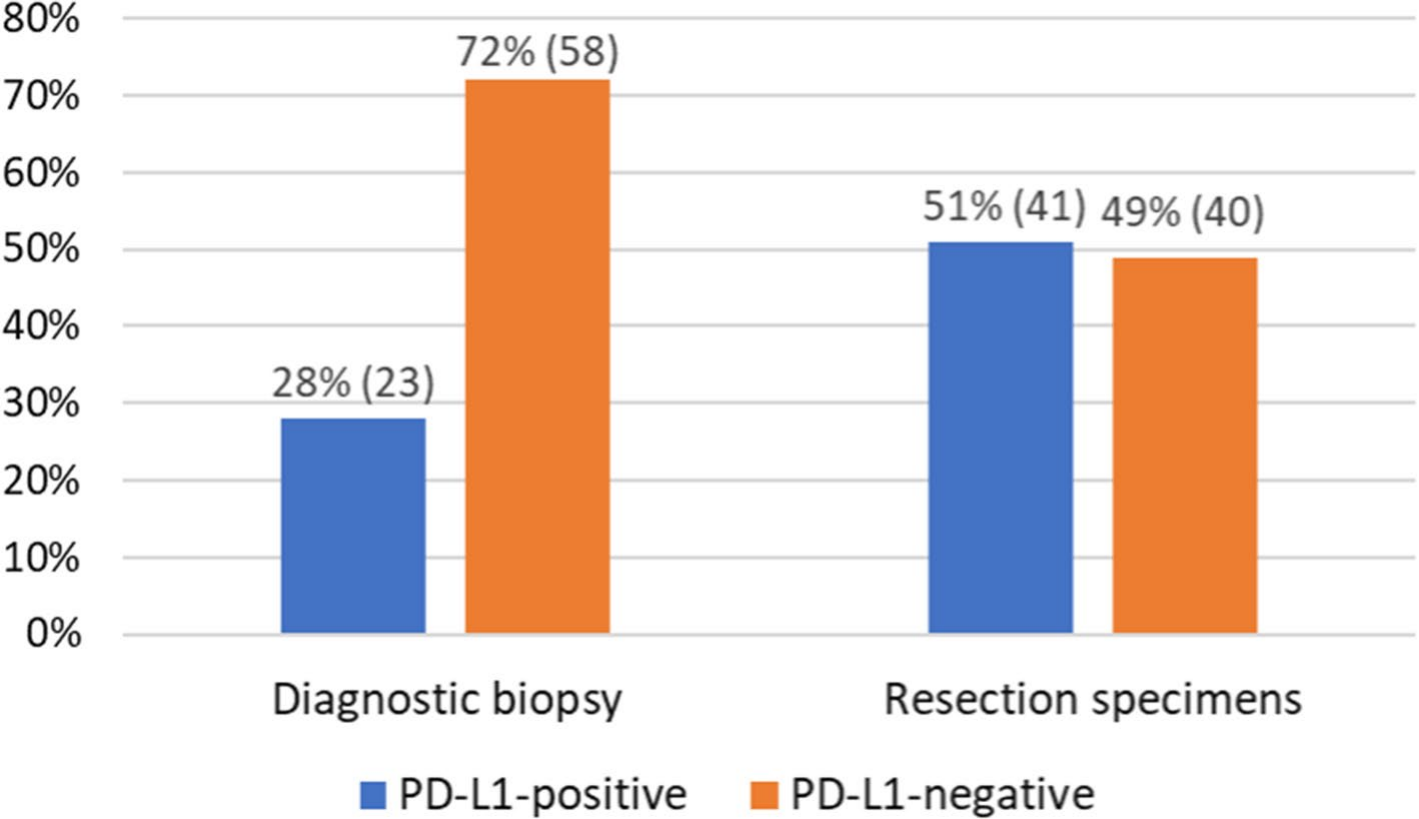
The distribution of PD-L1 status among diagnostic biopsies and matched surgically resected specimens

Firstly, we evaluated 81 tissue samples from preoperative breast biopsies. Twenty-three (28%) TNBC biopsy specimens were PD-L1-positive, while 58 (72%) were PD-L1-negative. In 17 (21%) PD-L1-negative tumors, PD-L1 expression covered less than 1% of the tumor area, while no PD-L1 expression was found in 41 biopsy specimens (51%) (Fig. 1).

Secondly, we evaluated PD-L1 expression in 58 surgically resected tissue specimens matching the primarily PD-L1-negative biopsies. Eighteen (31%) resection specimens, primarily classified as PD-L1-negative, were determined to be positive during postoperative examination. The remaining 40 resection specimens were PD-L1-negative. Among the PD-L1-negative specimens, in 10 (17%), the expression of PD-L1 covered less than 1% of the tumor area, and 31 (53%) specimens completely lacked PD-L1 expression.

**Fig. 2:**
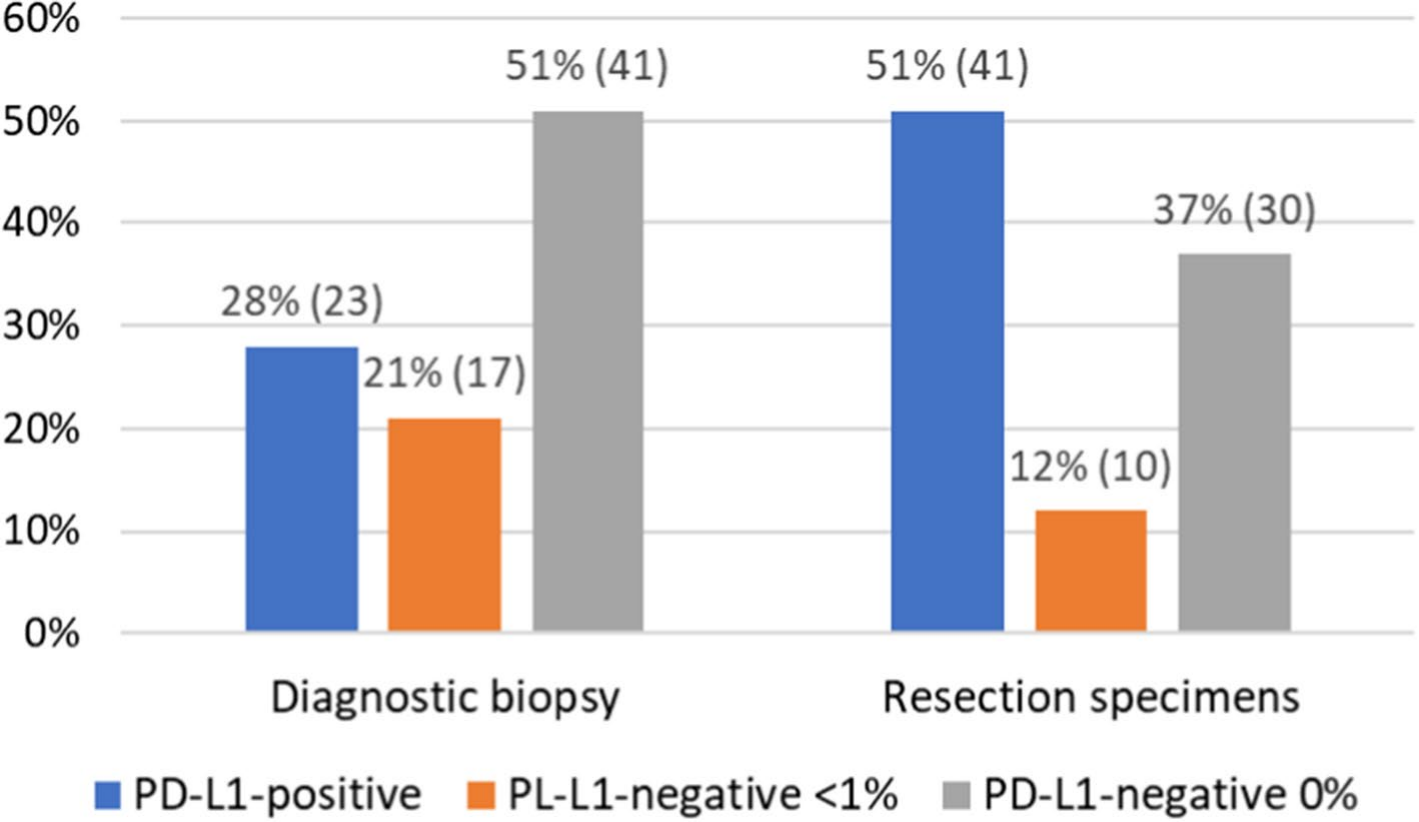
Detailed distribution of PD-L1 expression among primary PD-L1-negative patients in diagnostic biopsy and reevaluated matched resected tumor. Specimens with IC ≥ 1% were considered PD-L1-positive. All specimens with IC < 1% were PD-L1-negative. Some samples showed a slight PD-L1 staining, which did not exceed 1% IC. Those samples were labeled as PD-L1-negative < 1%

Considering the pre- and postoperative analyses, we found 41 (51%) PD-L1-positive tumors and 40 (49%) PD-L1-negative tumors (Fig. 1). In 10 (12%) PD-L1-negative specimens, the expression of PD-L1 was found in less than 1% of the tumor area, and 30 (37%), we observed no signs of PD-L1 expression (Table 1). We found 18 (22%) more PD-L1-positive TNBCs in surgical than in biopsy specimens, and the difference was statistically significant (*p* = 0.0038) (Fig. 2). For all cases when the biopsy was considered positive for PD-L1 staining, the PD-L1 status was confirmed in resection specimens, and no false positives were found. The correlation between the results of TNBC biopsies and resection specimen analyses (*k* = 0,495; *p* = 0.0008) was statistically significant. The agreement rate between biopsy and resection specimen results was 77.77%, with Cohen’s Kappa of 55.79%.

### The analysis of PD-L1 staining patterns and TNBC histopathological features

**Fig. 3:**
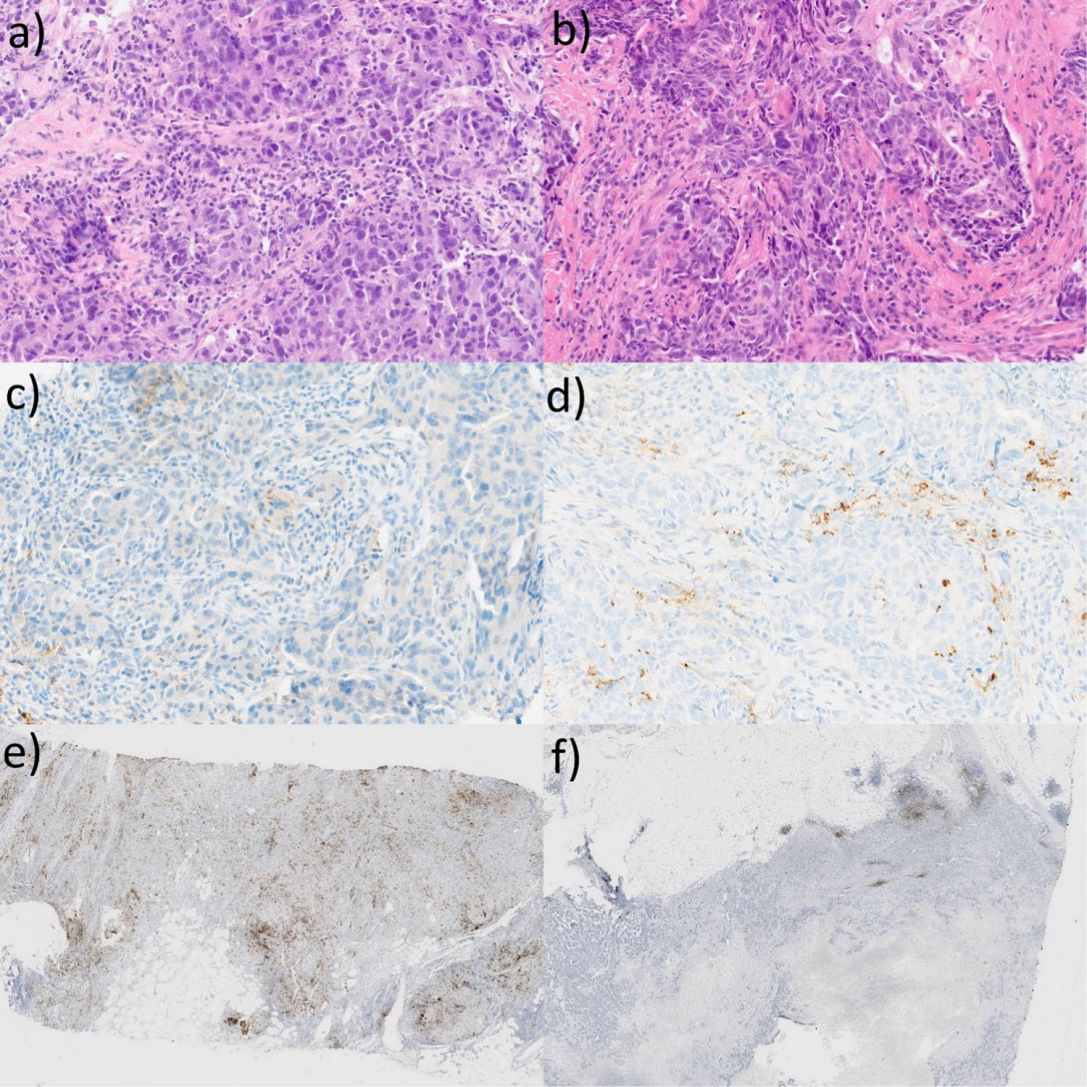
Cross-section images of **a** TNBC with marked features of inflammation; **b** TNBC without marked signs of inflammation; **c** PD-L1 in TNBC with marked features of inflammation; **d** PD-L1 in TNBC without marked signs of inflammation; **e** “dispersed” PD-L1 staining in TNBC; **f** “aggregated” PD-L1 staining in TNBC; TNBC—triple-negative breast cancer; and PD-L1—programmed cell death ligand 1

To investigate the cause of found discrepancies in PD-L1 expression between biopsy and resection specimens, we analyzed the staining patterns of PD-L1 expression in the resection specimens, the intensity of inflammation in the tumor stroma, and the correlation between the PD-L1 status of a tumor with clinical stage, grade, and lymph node involvement [Fig. 3].

In our cohort, PD-L1-positive immune cells showed two patterns of PD-L1 distribution—they formed aggregates in specific parts of the tumor (the “aggregated” pattern) or were dispersed through the specimen (the “dispersed” pattern). We found that out of 41 PD-L1-positive resection specimens, 13 had aggregated and 28 had dispersed PD-L1 staining patterns. The PD-L1 staining was more likely to be aggregated if the tumor was initially considered negative for PD-L1 based on the core biopsy analysis but turned out to be PD-L1-positive after the evaluation of the matched resection specimen. If the initial biopsy was PD-L1-positive, the PD-L1 expression was more likely to be dispersed (*p* = 0.0008).

Most tumors (67/81) showed signs of at least mild inflammation, determined as the presence of a cluster of inflammatory cells or at least > 10 tumor-infiltrating lymphocytes per high-power field. In the other 14 tumors, we found no or occasional single inflammatory cells. PD-L1 positivity was associated with more severe inflammation (*p* = 0.0001, *k* = 0,50).

We observed no correlation between the tumor PD-L1 status, clinical stage, grade, or lymph node involvement (*p* > 0.05). 

## Discussion

We found significantly more PD-L1-positive TNBCs among resection specimens than biopsies (*p* < 0.05). After the postoperative analysis, 18 out of 58 tumors classified as PD-L1-negative turned out to be PD-L1-positive. Our study reveals a significant discordance in the PD-L1 status between TNBC core biopsy samples and matched surgical specimens. Only slightly more than half of all PD-L1-positive cases (18/41; 56%) were identified by biopsy, indicating that the single diagnostic biopsy specimens are unrepresentative of the whole tumor PD-L1 status.

### Discrepancies in assessing PD-L1 status in TNBC

Only a few studies have addressed the role of PD-L1 heterogeneity in tumor misclassification. Noske et al. examined PD-L1 expression in TNBC using VENTANA SP142, VENTANA SP263, and DAKO 22C3 assays [12]. PD-L1 positivity was defined as > 1% of the percentage of tumor area covered by tumor-infiltrating immune cells (IC) and when the percentage of all stained cells in relation to all viable cells in a sample was > 1%. PD-L1 positivity for IC and CPS (Combined Positive Score) varied between assays and reached 11.1% for SP142 and 61.1% for SP263. The concordance rates between biopsies and resection specimens were 78% for SP263, 72% for 22C3, and 54% for SP142. No significant correlation existed between the PD-L1 status of biopsies and surgical specimens assessed by the SP142 test [12]. Hence, SP142 may not be the most suitable to assess PD-L1 expression in TNBC.

Dobritoiu et al. assessed the PD-L1 status in TNBC using the SP142 protocol and reported that among the analyzed biopsy specimens, 22 (30%) were PD-L1-positive and 51 (70%) PD-L1-negative [13]. Similarly to our research, upon examining the resection specimens, another 16 tumors came to be PD-L1-positive, while none of the initially PD-L1-positive tumors was reclassified as negative. Overall, 22% of all patients would be misclassified as PD-L1-negative and not receive immune checkpoint therapy if their PD-L1 status had been assessed by biopsy only [13]. In clinical audit data of 1458 TNBC samples, 490 (33.6%) were originally considered positive, but after retesting, an additional 58 (5.3%) samples turned out to be positive [13].

Dori et al. stratified the risk of PD-L1 status misclassification in TNBC using a computer model that calculated the impact of the staining intensity and distribution on diagnostic accuracy [14]. A higher risk of misclassification was associated with larger PD-L1 aggregates, smaller biopsy samples, and higher intra-tumor heterogeneity. Samples in which the estimated PD-L1 expression was near the cutoff also had a higher risk of error. Surprisingly, the false positives in their study were higher than the false negatives, showing a trend contradictory to other literature reports [12–14].

The results of our analysis seem in line with the literature reports, indicating that the pattern of PD-L1 distribution limits the accuracy of core biopsies [14]. Tumors in which PD-L1-positive cells form aggregates are more likely to be misclassified as PD-L1-negative due to a large tumor area with a relative lack of PD-L1 expression. On the other hand, dispersed PD-L1 staining was more easily detectable, and such tumors were less likely to be misclassified by biopsy (*p* < 0.05) [Fig. 3].

### Refining PD-L1 assessment in TNBC

Given that the IHC-based detection of PD-L1 does not always reflect patients’ response to anti-PD-1/PD-L1 therapy, multiple methods to improve the testing accuracy in TNBC have been proposed.

Ho Baek et al. performed SP142 assays twice for biopsy and surgery specimens collected from 77 early TNBC patients. 68.8% of specimens were considered PD-L1-positive after the second examination compared to 37.6% after the initial test [15]. Khan et al. also observed in-tumor PD-L1 expression heterogeneity, reflected by the lack of agreement between the results of different biopsy samples of the same tumor [16]. Multiple core biopsies could better reflect the PD-L1 expression within the entire tumor. Still, prior examination of the PD-L1 expression pattern in TNBC and randomized clinical trials are required to assess their clinical utility.

Paré et al. recently examined PD-1 mRNA expression in 10,078 samples of 34 different cancers and found a significant correlation between PD-1 mRNA and the overall response rates to anti-PD-1 monotherapy. In the same study, PD-L1 tumor expression assessed by IHC was not associated with patients’ responses [17].

Other emerging biomarkers include microsatellite instability (MSI), tumor mutational burden (TMB), mismatch repair (MMR) deficiency, tumor-infiltrating lymphocytes (TILs), gene signatures, and oncogene mutations [2, 18, 19]. However, whether they could outperform the PD-L1-based tests in predicting treatment efficacy remains unclear.

### Clinical perspectives

IHC-based PD-L1 assessment remains the most prevalent method of predicting patient’s response to immune checkpoint inhibitors. Presently, pembrolizumab in combination with chemotherapy is approved for treating patients with locally recurrent unresectable or metastatic TNBC expressing PD-L1 (CPS ≥ 10) [20]. The FDA approved the PD-L1 IHC 22C3 protocol to standardize the measurement to test PD-L1 expression in TNBC [21].

On the other hand, atezolizumab, an anti-PD-L1 antibody, has been approved by the European Medicines Agency (EMA) to treat patients with metastatic or unresectable TNBC that have not received prior chemotherapy and whose tumors have ≥ 1% of PD-L1 expression [22]. While no IHC test is currently recommended, SP142 was used to test for PD-L1 in TNBC before the FDA withdrew its approval in 2021. It was a result of the IMpassion131 trial that failed to meet the primary endpoint of PFS superiority in the frontline treatment of patients with PD-L1 positivity (HR, 0.82; 95% CI, 0.60–1.12; *P* = 0.20). There was no difference in survival advantage in the PD-L1-positive (HR 1.11, 95% CI 0.76–1.64). Even though the Oncology Drugs Advisory Committee (ODAC) voted 7 to 2 to maintain the accelerated approval of atezolizumab in April 2021, the FDA has withdrawn approval for atezolizumab in TNBC [23].

The rationale for targeting the PD-1/PD-L1 pathways in TNBC requires further validation. Enhancing the accuracy of PD-L1 assessment appears to be pivotal for adequate patient therapy. A great effort is put into standardizing diagnostic procedures and sample assessment. For instance, a sample is considered adequate and analyzed if it contains at least 50 viable tumor cells with associated stroma [24]. Data regarding sampling criteria in TNBC still need to be included. While some conclusions can be extrapolated from other cancers—like the need to ensure sufficient sample size (> 2 cm) or the effects of PD-L1 glycosylation before staining and analysis—their adaptation to TNBC protocols requires further research [25, 26].

## Conclusions

Single biopsy does not accurately reflect the PD-L1 expression in TNBC. Our results suggest that a significant subset of TNBC patients may be misclassified as PD-L1-negative and disqualified from anti-PD-L1 therapy. It remains to be elucidated whether multiple biopsies, additional PD-L1 tests, PD-L1 pre-analytic processing, or alternative predictive markers could improve the outcomes of initially PD-L1-negative patients.

## Data Availability

The datasets generated during and/or analyzed during the current study are available from the corresponding author upon reasonable request.
